# Prevalence of chikungunya virus infection in Sabah, Malaysia during 2017–2020

**DOI:** 10.1186/s41182-025-00735-3

**Published:** 2025-04-21

**Authors:** Mya Myat Ngwe Tun, Jecelyn Leaslie John, Thanh Vu Nguyen, Maurine Mumo Mutua, Abdul Marsudi Manah, Yuki Takamatsu, Takeshi Urano, Kouichi Morita, Kamruddin Ahmed

**Affiliations:** 1 Department of Tropical Viral Vaccine Development, Institute of Tropical Medicine, Nagasaki University, Nagasaki, Japan; 2 Department of Tropical Viral Vaccine Development and Department of Virology, Institute of Tropical Medicine, Nagasaki University, Nagasaki, 852-8523 Japan; 3 Center for Vaccines and Therapeutic Antibodies for Emerging Infectious Diseases, Shimane University, Izumo, 690-8504 Japan; 4 Borneo Medical and Health Research Centre, Faculty of Medicine and Health Sciences, Universiti Malaysia Sabah, Kota Kinabalu, Sabah Malaysia; 5 Pasteur Institute in Ho Chi Minh City, Ho Chi Minh City, Vietnam; 6 Sabah States Health Department, Kota Kinabalu, Sabah Malaysia; 7 DEJIMA Infectious Diseases Research Alliance, Nagasaki University, Nagasaki, Japan; 8 Department of Pathology and Microbiology, Faculty of Medicine and Health Sciences, Universiti Malaysia Sabah, 88400 Kota Kinabalu, Sabah Malaysia; 9 Tropical Medicine and Health, Nagasaki University, Nagasaki, Japan

**Keywords:** CHIKV, Real time PCR, Neutralization test, Sabah

## Abstract

**Background:**

Chikungunya virus (CHIKV) is a mosquito-borne alphavirus and an emerging global health threat. Most research on CHIKV in Malaysia has primarily focused on Peninsular Malaysia, with limited data on its distribution in the endemic regions of Borneo, particularly Sabah. This study aimed to determine the prevalence of CHIKV infection in Sabah, Malaysia.

**Methods:**

A total of 130 serum samples, comprising 74 from febrile patients and 56 healthy individuals were collected between 2017 and 2018. Subsequently, 188 serum samples were obtained from febrile patients in Sabah, Malaysia during 2019–2020. All samples underwent quantitative real-time reverse transcription polymerase chain reaction (RT-qPCR) for the detection of the CHIKV genome. Additionally, serological tests were conducted to identify anti-CHIKV IgM and IgG antibodies. Serologically positive samples were further validated using neutralization assays to confirm the presence of CHIKV-specific antibodies.

**Results:**

In 2017–2018, 16 out of 130 samples (12.3%) tested positive for recent CHIKV infections based on CHIKV RT-qPCR or anti-CHIKV IgM results, while in 2019–2020, 7 out of 188 samples (3.7%) showed recent infections. Among the 16 recent CHIKV-positive cases in 2017–2018, four were asymptomatic individuals. In 2017–2018, 24 (18.4%) individuals tested positive for anti-CHIKV IgG, with 15 (11.5%) showing positive neutralization test results. In 2019–2020, 20 (10.6%) febrile patients were seropositive for anti-CHIKV IgG, with 17 (9.0%) showing CHIKV neutralization positivity. The CHIKV infection rate in Sabah was higher during 2017–2018 compared to 2019–2020.

**Conclusions:**

To the best of our knowledge, this is the first report confirming the presence of CHIKV in both patients and healthy individuals in Sabah using RT-qPCR and neutralization tests. Although the likelihood of transmission from asymptomatic individuals is low, they still present a considerable public health risk. Our results indicate that both basic scientists and clinicians should consider CHIKV when diagnosing febrile patients, and policymakers should put in place effective surveillance and control measures.

## Introduction

Chikungunya virus (CHIKV) infection has emerged as a global health threat over the past two decades, spreading worldwide and infecting millions in Asia, Africa, Europe, the Americas, and the Pacific Islands. The virus is transmitted to humans by infected mosquitoes through a human-mosquito-human bite [1]. The clinical presentation of CHIKV disease varies from self-limiting undifferentiated febrile illness to debilitating polyarthritis and encephalitis and, in some cases, death [2]. Approximately 3–28% of people infected with CHIKV will remain asymptomatic. For people who develop symptomatic illness, the incubation period is typically 3–7 days (range 1–12 days) [2, 3]. The diagnostic challenge of CHIKV arises from its clinical resemblance to other arboviruses, especially dengue virus (DENV) and Zika virus (ZIKV), which belong to the *Flavivirus* genus and the *Flaviviridae* family and often circulate together in endemic areas [2, 4]. Three diseases cause similar presentations, with fever, myalgia, headache, arthralgia, and rash; however, acute arthritis and rash are more prominent in CHIKV infection.

Malaysia is a tropical country consisting of two regions, West Malaysia (Peninsular) and East Malaysia (Sabah, Sarawak, and the Federal Territory of Labuan) separated by the South China Sea*.* Both *Ae. aegypti* and *Ae. albopictus* are widespread in Malaysia, which continues to grapple with the ongoing threat of mosquito-borne diseases such as dengue, chikungunya, and Zika. Malaysia encountered CHIKV infection with a low seropositivity level in 1960, followed by limited outbreaks in 1998 [5]. In 2006, a localized CHIKV outbreak occurred in Bagan Pachor, caused by the Asian genotype [6]. A larger nationwide outbreak took place between 2008 and 2010, driven by the East-Central-South-Africa (ECSA) genotype [7, 8]. Since then, CHIKV has remained a threat in Malaysia, with small outbreaks in 2017 due to the reemergence of the ECSA genotype [9]. In 2020, a concerning surge in CHIKV cases was reported in Perak, Malaysia [10]. Most studies on CHIKV have been reported from Peninsular Malaysia; however, distribution data from endemic regions of Borneo, especially Sabah, are very limited [11]. In this study, we aimed to determine the prevalence of CHIKV infection among febrile patients and healthy persons in Sabah, Malaysia in 2017–2020.

## Materials and methods

**Fig. 1:**
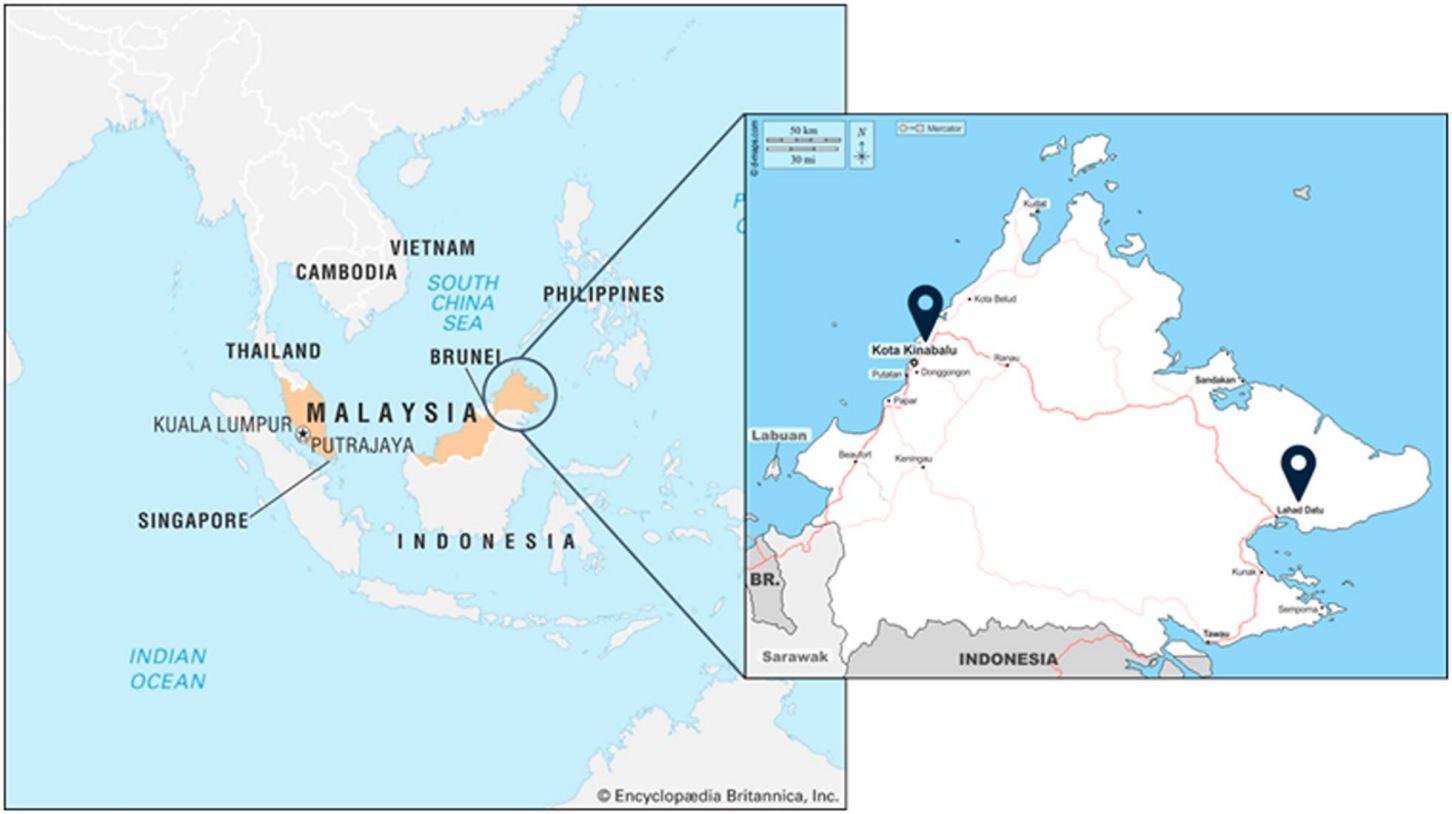
Map of Kota Kinabalu and Lahad Datu in Sabah, Malaysia where acute serum samples were collected during 2017–2020

Between 2017 and 2018, serum samples were collected from 74 febrile patients at Kota Marudu Hospital and 56 healthy volunteers at Queen Elizabeth II Hospital in Kota Kinabalu City, Sabah. From 2019 to 2020, 188 acute serum samples of febrile patients from Kota Kinabalu and Lahad Datu were collected from Kota Kinabalu Public Health Laboratory in Sabah, Malaysia (Fig. 1). The samples from febrile patients and healthy volunteers were collected under the same conditions as blood collection tube type, same period of sample collection (April-November) and same location (Kota Kinabalu). Serological confirmation of CHIKV infection was carried out using an in-house IgM capture ELISA, as previously outlined [12]. A sample was considered positive if the optical density (OD) ratio of the positive control (or test sample) to the negative control was equal to or greater than 2.0 [12]. To measure anti-CHIKV IgG levels in serum samples, an in-house anti-CHIKV IgG indirect ELISA was used as per our previous protocol [12] and a sample was considered positive if the titer was equal to or greater than 1:3000. To further confirm the presence of specific antibodies in CHIKV ELISA-positive samples, serum samples were tested for their ability to neutralize CHIKV using a 50% focus reduction neutralization test (FRNT_50_), as previously described, with an NT cutoff value of 1:10 [12].

To detect the CHIKV genome, RNA was extracted from serum samples using the QIAamp Viral RNA Mini Kit (QIAGEN, Hilden, Germany). A two-step real-time reverse transcription-quantitative polymerase chain reaction (RT-qPCR) was then performed, based on a previously established protocol [13]. Following reverse transcription with the PrimeScript^™^ RT Reagent Kit (Takara, Shiga, Japan), real-time PCR was performed using SYBR^®^ Premix Ex Taq^™^ II (Tli RNaseH Plus, Takara, Shiga, Japan), in accordance with the manufacturer’s guidelines. A cycle threshold (Ct) value of less than 37 was considered indicative of a positive result for CHIKV in the RT-qPCR.

## Results

Out of the 74 and 56 serum samples collected from febrile patients and healthy individuals, respectively, in 2017–2018, three (4.1%) and one (1.8%) tested positive for CHIKV by RT-qPCR, while 10 (13.5%) and three (5.4%) were positive for anti-CHIKV IgM (Table 1). Among the three CHIKV RT-qPCR-positive febrile patients, one was also positive for anti-CHIKV IgM. Two samples each from anti-CHIKV IgM-positive febrile patients and healthy individuals showed positivity for anti-CHIKV IgG (Table 1). In 2019–2020, out of 188 acute serum samples from febrile patients, two (1.1%) and five (2.7%) tested positive for CHIKV by RT-qPCR and anti-CHIKV IgM, respectively (Table 1). Among the five anti-CHIKV IgM-positive samples, four samples were anti-CHIKV IgG-positive. In Sabah, Malaysia, 12.3% (16/130) of cases from 2017 to 2018 and 3.7% (7/188) from 2019 to 2020 were found to have had recent CHIKV infections, as indicated by positive CHIKV RT-qPCR or anti-CHIKV IgM results.

**Table 1:** Recent chikungunya virus infection among febrile patients and healthy individuals in Sabah, Malaysia during 2017–2020

Sample collection year	Sample ID	CHIKV			
		IgM ratio	IgG titer	NT	Viral RNA copies/ml
2017–2018	S-1	**3.3**	1388	< 10	**3.20E + 04**
	S-2	1.0	**3380**	< 10	**8.80E + 03**
	S-3	0.8	994	< 10	**8.80E + 03**
	S-4	**3.3**	1388	< 10	Undetermined
	S-5	**3.3**	2967	< 10	Undetermined
	S-6	**4.6**	1109	< 10	Undetermined
	S-7	**4.0**	1013	ND	Undetermined
	S-8	**3.4**	804	< 10	Undetermined
	S-9	**3.6**	498	< 10	Undetermined
	S-10	**5.2**	433	ND	Undetermined
	S-11	**2.4**	**3385**	< 10	Undetermined
	S-12	**2.2**	**11,154**	**20**	Undetermined
2017–2018	H-1	0.8	1128	< 10	**3.20E + 04**
	H-2	**3.4**	484	< 10	Undetermined
	H-3	**2.2**	**3304**	< 10	Undetermined
	H-4	**2.1**	**20,502**	**40**	Undetermined
2019–2020	S-13	0.8	319	< 10	**2.40E + 04**
	S-14	0.6	302	< 10	**1.60E + 04**
2019_2020	S-15	**3.7**	601	< 20	Undetermined
	S-16	**3.6**	**9696**	**160**	Undetermined
	S-17	**2.9**	**12,638**	**160**	Undetermined
	S-18	**3.6**	**9696**	**160**	Undetermined
	S-19	**2.2**	**4546**	**80**	Undetermined

CHIKV; chikungunya virus, S; febrile patient’s sample, H; healthy person’s sample, ND; not done due to serum shortage, undetermined; quantitative real time RT-PCR (RT-qPCR) negative, NT; neutralization titer, Bold font; positive for IgM (≥ 2), IgG (≥ 3000), NT (≥ 10) and RT-qPCR (excluding undetermined cases)

**Fig. 2:**
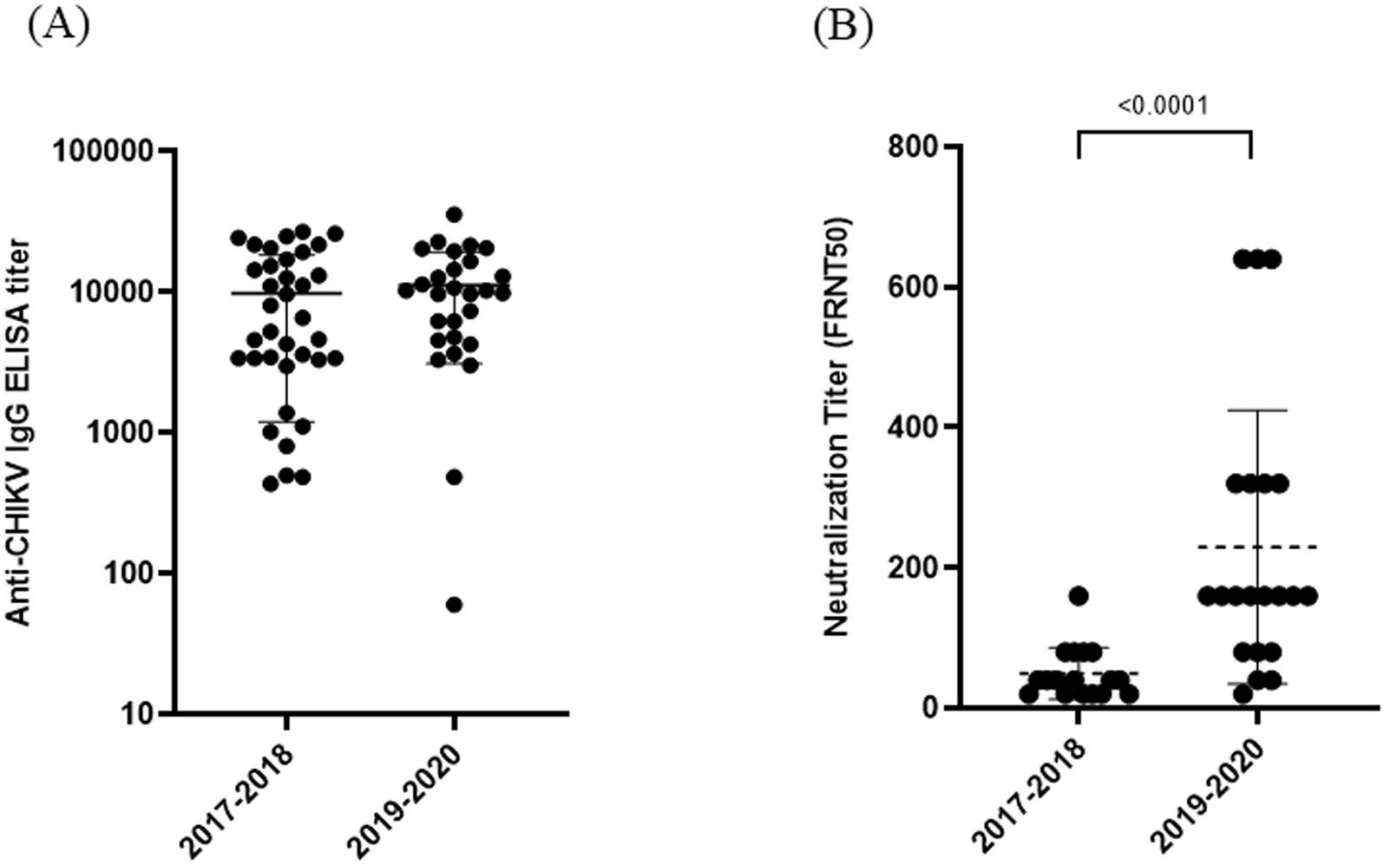
**(A)** Anti-CHIKV IgG titer and **(B)** neutralization titer against CHIKV in febrile patients and healthy individuals between 2017–2018 and 2019–2020. The mean of CHIKV-neutralizing antibodies was compared by period years using the Mann–Whitney U test

To assess past CHIKV infections, we measured anti-CHIKV IgG levels in the serum of both patients and healthy individuals. Of the 74 serum samples from febrile patients and 56 from healthy individuals collected in 2017–2018, 19 (25.7%) and five (8.9%) were seropositive for anti-CHIKV IgG, respectively. Among these anti-CHIKV IgG ELISA-positive individuals, 12 (16.2%) febrile patients and three (5.4%) healthy individuals tested positive for CHIKV neutralization (Table 2). In total, 18.4% (24/130) of samples were seropositive for anti-CHIKV IgG and 11.5% (15/130) for neutralization in 2017–2018. In 2019–2020, out of 188 acute serum samples from febrile patients, 20 (10.6%) were seropositive for anti-CHIKV IgG, and 17 (9.0%) of the anti-CHIKV IgG ELISA-positive samples tested positive for CHIKV neutralization (Table 2). The anti-CHIKV IgG titer in febrile patients/healthy individuals from 2017 to 2018 did not differ significantly from the IgG titer observed in febrile patients in 2019–2020 (Fig. 2A). However, the neutralization titer against CHIKV in patients from 2019 to 2020 was significantly higher than that in febrile patients/healthy individuals from 2017 to 2018 (Fig. 2B).

**Table 2:** Past chikungunya virus infection among febrile patients and healthy individuals in Sabah, Malaysia during 2017–2020

Sample collection year	Sample ID	CHIKV		
		IgM ratio	IgG titer	NT
2017–2018	S-1	1.3	**26,864**	**160**
	S-2	1.7	**21,691**	**20**
	S-3	0.7	**13,090**	**40**
	S-4	1.2	**4612**	< 10
	S-5	1.5	**14,383**	**40**
	S-6	1.0	**3385**	< 10
	S-7	0.9	**3435**	< 10
	S-8	1.0	**3380**	< 10
	S-9	0.8	**8028**	**20**
	S-10	1.0	**4557**	ND
	S-11	1.6	**24,274**	**80**
	S-12	1.6	**24,857**	**80**
	S-13	1.3	**17,051**	**40**
	S-14	1.1	**9610**	**20**
	S-15	0.7	**3590**	< 10
	S-16	1.2	**15,343**	**20**
	S-17	0.8	**25,995**	**40**
	S-18	1.5	**11,024**	< 10
	S-19	0.8	**12,577**	**40**
2017–2018	H-1	0.8	**5239**	< 10
	H-2	0.8	**6550**	**20**
	H-3	0.7	**21,681**	**80**
	H-4	0.9	**4270**	< 10
	H-5	0.8	**19,198**	**80**
2019–2020	S-20	1.7	**16,516**	**160**
	S-21	0.7	**9828**	**160**
	S-22	1.0	**3654**	**40**
	S-23	0.8	**22,709**	**640**
	S-24	0.8	**3020**	< 10
	S-25	1.1	**35,655**	**640**
	S-26	1.0	**12,898**	**320**
	S-27	1.1	**10,225**	**160**
	S-28	0.8	**6194**	**160**
	S-29	1.5	**10,225**	ND
	S-30	1.0	**6194**	ND
	S-31	0.5	**21,511**	**320**
	S-32	0.8	**14,410**	**320**
	S-33	1.8	**4775**	**40**
	S-34	0.8	**20,313**	**320**
	S-35	0.9	**11,337**	**160**
	S-36	1.6	**10,682**	**80**
	S-37	0.7	**19,482**	**640**
	S-38	0.4	**4247**	**20**
	S-39	0.7	**7313**	**80**

CHIKV; chikungunya virus, S; febrile patient’s sample, H; healthy person’s sample, ND; not done due to serum shortage, negative, NT; neutralization titer, Bold font; positive for IgG, NT

## Discussion

The spread of CHIKV infections have been observed in various regions globally [12, 14, 15]. In certain areas, such as Malaysia, CHIKV outbreaks are typically sporadic, occurring on a smaller scale and being localized [16–19]. It is still unclear whether this limitation is due to the small sample size of studies or the under-reporting of cases. In this study, we reported the prevalence of recent and past CHIKV infections among febrile patients and healthy individuals from 2017 to 2020 in Sabah, Malaysia. Since 2019, Malaysia has experienced a rise in CHIKV infections, indicating a resurgence of the disease after the previous major outbreak in 2008 [20]. There has also been a notable shift in the geographical distribution of cases, with the focus increasingly moving from predominantly rural areas between 2009 and 2011 toward concentration in urban regions from 2017 to 2022 [9]. Most of these cases were reported in Peninsular Malaysia, while in 2019–2020, the Ministry of Health Malaysia documented only one case of CHIKV infection in Sabah [21].

In our study, 12.3% of cases from 2017 to 2018 and 3.7% from 2019 to 2020 were found to have had recent CHIKV infections. Notably, among the recent CHIKV infection cases in 2017–2018, 7.1% were from healthy individuals who showed no symptoms of the infection. The rate of past CHIKV infections in healthy individuals was 5.4% in 2017–2018, which aligns closely with findings from previous studies that reported a rate of 5.9% in healthy adults in Peninsular Malaysia [5]. Additionally, CHIKV antibodies confirmed by neutralization tests were found in 16.2% of febrile patients in 2017–2018 and 9% in 2019–2020.

To the best of our knowledge, this is the first report confirming the presence of CHIKV in both patients and healthy individuals in Sabah using RT-qPCR and neutralization tests. Asymptomatic infections in healthy individuals serve as silent drivers of pandemics. While the risk of transmission from asymptomatic persons is low, they may still pose a significant public health threat. Our findings suggest that basic scientists and clinicians should include CHIKV in the differential diagnosis of febrile patients, and policymakers should implement appropriate surveillance measures and control strategies.

## Data Availability

All data generated or analyzed during this study are included in this published article and its supplementary information file.

## Abbreviations

CHIKV: Chikungunya Virus
RT-PCR: Reverse Transcriptase-Polymerase Chain Reaction
RT-qPCR: Quantitative real time RT-PCR
IgM: Immunoglobulin M
IgG: Immunoglobulin G
DENV: Dengue virus
ZIKV: Zika virus
ECSA: East Central South Africa
ELISA: Enzyme Linked Immunosorbent Assay
FRNT_50_: 50% Focus reduction neutralization test
